# Somatosensory evoked high-frequency oscillations and prognostication after cardiac arrest

**DOI:** 10.1186/cc14511

**Published:** 2015-03-16

**Authors:** C Endisch, C Storm, CJ Ploner, C Leithner

**Affiliations:** 1 Charité Universitätsmedizin Berlin, Germany

## Introduction

Electrical median nerve stimulation elicits a burst of high-frequency oscillations (HFOs) superimposing onto the cortical short-latency potential. Digital filtering of somatosensory evoked potentials (SSEPs) enables non-invasive analysis of these HFOs. The late HFO component following the cortical N_2_O peak is ascribed to spiking activity of cortical neurons.

## Methods

We retrospectively studied late HFO components of median nerve SSEPs obtained 24 hours to 4 days after cardiac arrest in patients treated with mild hypothermia (33°C for 24 hours). Cortical average recordings were digitally filtered at 450 to 750 Hz and noise-corrected maximum peak-to-peak amplitudes of the cortical late HFO bursts determined. Outcome upon ICU discharge was dichotomized according to the Cerebral Performance Category (CPC) scale. CPC 1 to 3 was classified as good outcome, CPC 4 to 5 (unresponsive wakefulness syndrome and death) as poor outcome.

## Results

Of 307 consecutive patients, 153 (50%) achieved good outcome (CPC 1 to 3) and 154 (50%) had poor outcome. Late HFO bursts were present in 102 (33%) recordings. Among patients with late HFO amplitudes above 0.1 μV, 26 had CPC 1 to 3, none had CPC 4 and eight died. Case review indicated causes of death other than hypoxic encephalopathy in all patients who died despite HFO amplitudes above 0.1 μV.

## Conclusion

We found cortical late HFO bursts, obtained by digital filtering of standard SSEP recordings, in a significant proportion of patients after cardiac arrest treated with mild hypothermia. Our data indicate that the presence of late HFO bursts with amplitudes above 0.1 μV may confirm absence of severe hypoxic encephalopathy early after cardiac arrest with high specificity.

